# High Expression of COL10A1 Is an Independent Predictive Poor Prognostic Biomarker and Associated with Immune Infiltration in Advanced Gastric Cancer Microenvironment

**DOI:** 10.1155/2022/1463316

**Published:** 2022-10-13

**Authors:** Neng Shen, Shisheng Zhu, Zhongyan Zhang, Xuejiao Yong

**Affiliations:** ^1^Department of Gastroenterology, Chongqing University Cancer Hospital, Chongqing, China; ^2^Faculty of Basic Medical Sciences, Chongqing Medical and Pharmaceutical College, Chongqing 401331, China; ^3^Oncology Treatment Center of Traditional Chinese Medicine, Chongqing University Cancer Hospital, Chongqing 400030, China

## Abstract

Medical technology has become more and more sophisticated recently, which, however, fails to contribute to a better prognosis for patients suffering advanced gastric cancer (GC). Hence, new biomarkers specific to GC diagnosis and prognosis shall be identified urgently. This study screened differentially expressed genes (DEGs) between 375 GC samples and 32 paracancer tissue samples from TCGA datasets. The expression of Collagen type X alpha 1 (COL10A1) in GC was analyzed. The chi-square test assisted in analyzing the relevance of COL10A1 to the clinicopathologic characteristics. The Kaplan-Meier method helped to assess the survival curves and log-rank tests assisted in the examination of the differences. The Cox proportional hazard regression model served for analyzing the risk factors for GC. Then, we developed a nomogram that contained the COL10A1 expression and clinical information. Finally, how COL10A1 expression was associated with the immune infiltration was also evaluated. In this study, 7179 upregulated and 3771 downregulated genes were identified. Among them, COL10A1 expression was distinctly increased in GC specimens compared with nontumor specimens. High COL10A1 expression exhibited an obvious relation to tumor T and pathologic stage. ROC assays confirmed the diagnostic value of COL10A1 expression in screening GC samples from normal samples. Survival data displayed that patients with high COL10A1 expression exhibited a shorter OS and DSS than those with low COL10A1 expression. We obtained a predictive nomogram, which could better predict the COL10A1 expression by virtue of discrimination and calibration. The prognostic value of COL10A1 expression was further confirmed in GSE84426 datasets. Immune assays revealed that COL10A1 expression was associated with tumor-filtrating immune cells, like CD8 T cells, cytotoxic cells, DC, eosinophils, iDC, macrophages, mast cells, NK CD56dim cells, NK cells, pDC, T helper cells, Tem, Th1 cells, Th17 cells, and Treg. Overall, we firstly proved that COL10A1 may be a novel and valuable prognostic and diagnostic factor for GC patients. In addition, COL10A1 has potential to be an immune indicator in GC.

## 1. Introduction

Gastric cancer (GC) is ranked fifth in incidence and fourth in mortality among numerous malignant tumors around the world [1]. Statistically, the year of 2015 saw 679,100 new GC cases and about 498,000 deaths caused by GC [2, 3]. Growing studies have proved the effect of personal lifestyle choices on GC, like inadequate fruits and vegetables, excessive drinking as well as high intake of salt [4, 5]. Besides, the risk of suffering GC can increase affected by a family history of GC and Helicobacter pylori infection [6, 7]. GC exhibits a low early diagnosis rate, and a majority of patients can only be diagnosed at an advanced stage; hence, its 5-year survival rate remains less than 10% [8, 9]. GC still exhibits poor prognosis despite the improvement made on the therapy methods, like chemotherapy, surgery, and targeted therapy [10]. On that account, it is suggested to confirm useful biomarkers for better assessing tumor development, predicting the overall survival (OS), and enhancing the treatment effects.

Collagen type X alpha 1 (COL10A1) belongs to a family of collagen [11]. COL10A1 gene is the alpha chain encoding form X collagen, the small chain collagen in the form of hypertrophic chondrocytes in the endochondral ossification process [12, 13]. It is a major matrix component in the stroma, and studies have confirmed the vital effect of extracellular matrix on tumor cells in terms of growth, differentiation, progression, apoptosis, and metastasis [14, 15]. A panel of RNAs prepared from various cancers and cancer cell lines were screened, finding the frequent upregulation of COL10A1 in various cancers. However, COL10A1 expression was limited or even could not be detected in a majority of normal tissues. COL10A1 can exhibit specific expression in the vasculature and tumor microenvironment for breast cancer tissues via the immunofluorescence staining by using specific antibodies [16]. The above findings met the results of another study. Huang et al. reported that colorectal cancer tissues showed obviously higher COL10A1 expression. As revealed by biological functional experiments, COL10A1 overexpression strengthened colorectal cancer cells in terms of the proliferation, the migration, and the invasion, and COL10A1 knockdown hindered the tumorigenesis in vivo. According to western blot assays, COL10A1 was capable of facilitating the epithelial-mesenchymal transition (EMT) process. In addition, serum protein concentrations regarding COL10A1 exhibited an obvious increase in adenomas and colorectal cancer cases relative to the control samples. It was possible to treat the protein level regarding COL10A1 in serum as a biomarker for diagnosing tumor prognosis in early stage, thereby identifying colorectal cancer and adenoma [17]. However, whether COL10A1 could be a diagnostic and prognostic biomarker for GC remained largely unclear.

In this study, we screened differentially expressed genes (DEGs) to compare GC specimens and nontumor specimens based on TGCA datasets and confirmed that COL10A1 expression showed an obvious increase in GC specimens. Then, we analyzed its diagnostic and prognostic value in two cohorts. Finally, the possible association of COL10A1 expression with immune microenvironment was explored.

## 2. Materials and Methods

### 2.1. Data Sources

The mRNA expression profiles regarding 375 GC samples and 32 paracancer tissue samples, together with related clinical data, came from The Cancer Genome Atlas (TCGA) database (https://www.cancer.gov/about-nci/organization/ccg/research/structural-genomics/tcga). 375 GC patients possessed matching mRNA expression profiles as well as survival data. Besides, the Gene Expression Omnibus (GEO) database (https://www.ncbi.nlm.nih.gov/geo/) provided the related dataset (GSE84426). The study strictly followed the publication guidelines of TCGA and GEO.

### 2.2. Differential Analysis of Genes

The “affy” and “limma” packages in R software (https://www.r-project.org/) served for differentiating specimens from the TCGA datasets, respectively, obtaining 375 GC samples and 32 paracancer tissue samples. The *t*-test assisted in screening DEGs following cut-off values: false discovery rate (FDR) < 0.05 and |log2fold change| > 1.

### 2.3. Analysis on the Correlation of COL10A1 Expression Patterns with Clinicopathological Features

We selected the clinicopathological data [age, gender, pathological stage, infiltration depth (T), distant metastasis (M), lymph node metastasis (N), etc.] regarding the RC tissue specimens in the TCGA database for later analysis. The study included clinical data of 367 patients except data that were defective or incomplete. An independent sample *t*-test together with a paired *t*-test assisted in confirming the correlation of COL10A1 expression with the clinical-pathological parameters.

### 2.4. Statistical Analysis on Potential Prognostic Factors

The R version 4.0.2 software (“survival” and “survminer” packages) served for identifying the potential prognostic factors. Univariate Cox regression analysis assisted in confirming many prognostic factors, and multivariate Cox regression analysis assisted in confirming independent prognostic factors. Nomograph was developed using R software. Finally, GSE84426 was used to validate the prognosis value of COL10A1 expression.

### 2.5. Protein Interaction Network Analysis

The STRING database (https://string-db.org/) served for exploring the predicted and actual correlations of protein interactions with COL10A1 expression patterns. Proteins interacting with the COL10A1 were screened.

### 2.6. Analysis on the Correlation of COL10A1 with Immune Cell Infiltration

The “cibersort” package (R version 4.0.2 software) assisted in analyzing the percentage occupied by 22 immune cell types (LM22 gene signature) in GC tissues. A further quantification was conducted on the correlation of COL10A1 expression with proportions occupied by different immune cells. The “ggplot2” and “limma” packages (R version 4.0.2 software) served for analyzing and plotting data at last. Also, we referenced the TIMER database for analyzing the tumor-infiltrating immune cells (CD8+ T cells, CD4+ T cells, B cells, macrophages, neutrophils, and DCs).

### 2.7. Gene Ontology (GO) and the Kyoto Encyclopedia of Genes and Genomes (KEGG) Pathway Analysis

GO enrichment that involves cellular components, molecular functions, and biological process is capable of defining special biological characteristics regarding certain genes in various respects. The KEGG enrichment served for investigating the biological pathways in some genes. GO and KEGG analysis were conducted under the assistance of the R 3.6 software together with “clusterProfiler” package. Moreover, “ggplot2” package was used for the outcome visualization.

### 2.8. GSEA

The median COL10A1 expression was taken into account for dividing patients into group with high expression and group with low expression by using the GSEA software; also, the gene enrichment pathways with the highest ranking in the two groups were detected (Molecular Signatures Database c2. Cp. Kegg. V7.2. Symbols). We used the Gene Matrix Transposed function dataset as a reference gene set specific to all analyses. FDR < 0.05 indicated significant enrichment.

### 2.9. Statistical Analysis

IBM SPSS Statistics for Windows, version 20.0 (IBM Corporation, Armonk, NY, USA) and R version 4.0.2 served for the statistical analyses. The gene expression data were in the form of mean ± standard deviation. A *t*-test assisted in comparing GC tissues and paracarcinoma tissues in terms of the COL10A1 expression in the TCGA and GEO databases. Wilcoxon's signed-rank test assisted in analyzing the correlation of the COL10A1 with clinical characteristic variables. The hazard ratio and 95% CI were calculated with the univariate and multivariate Cox analyses. Finally, R was used to draw nomogram and build a prediction model. We plotted ROC curve and calculated AUC by using “ROCR” package for assessing the ability to distinguish tumor and normal tissue. A *p* value < 0.05 reported statistical significance. FDR < 0.05 and *p* < 0.01 indicated significant enrichment.

## 3. Results

### 3.1. Microarray Data and Identification regarding DEGs in GC

**Figure 1:**
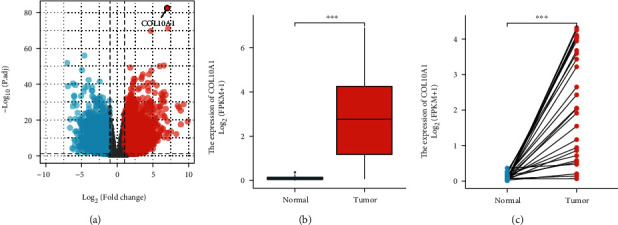
The COL10A1 expression in GC patients based on TCGA datasets. (a) Volcano plot served for visualizing the COL10A1 distribution of the DEGs. (b, c) Both paired and unpaired results found higher COL10A1 expression in tumor tissues relative to nontumor specimens. ^∗∗∗^*p* < 0.001.

For finding DEGs in GC, the current study included 375 GC samples and 32 paracancer tissue samples from the TCGA. At last, we identified 7179 upregulated and 3771 downregulated genes (|log FC| ≥ 1, p < 0.05). The COL10A1 distribution of the DEGs was visualized in a volcano plot (Figure 1(a)). Both paired and unpaired results displayed the higher COL10A1 expression in tumor tissues relative to control adjacent tissues (Figures 1(b) and 1(c)).

### 3.2. Relationship of Clinicopathological Characteristics with COL10A1 Expression

**Figure 2:**
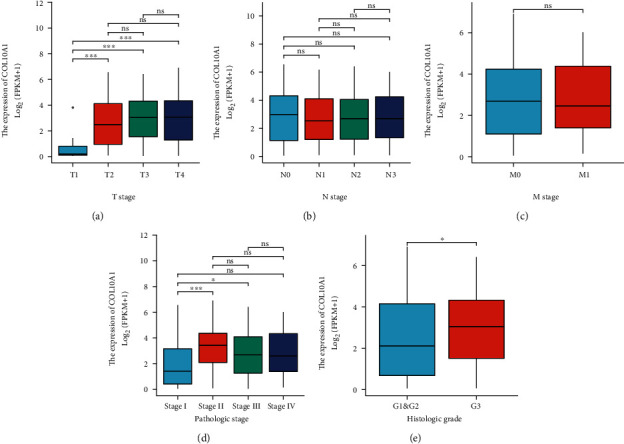
Relationship between clinicopathological characteristics and COL10A1 expression, namely (a) T stage, (b) N stage, (c) M stage, (d) pathologic stage, and (e) histologic grade. ^∗^*p* < 0.05,  ^∗∗^*p* < 0.01, and^∗∗∗^*p* < 0.001. ns: no significance.

**Figure 3:**
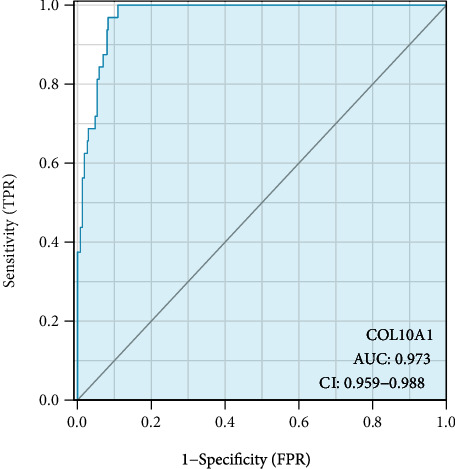
ROC curve of COL10A1 for the diagnosis of GC.

The clinical significances of COL10A1 expression were examined using the TCGA datasets. High COL10A1expression was significantly correlated with tumor T and pathologic stage (Table 1 and Figures 2(a)–2(e)). The level of COL10A1 can be used as a diagnosis tool for GC (AUC = 0.973) (Figure 3).

### 3.3. Prognosis Value of COL10A1 for GC

**Figure 4:**
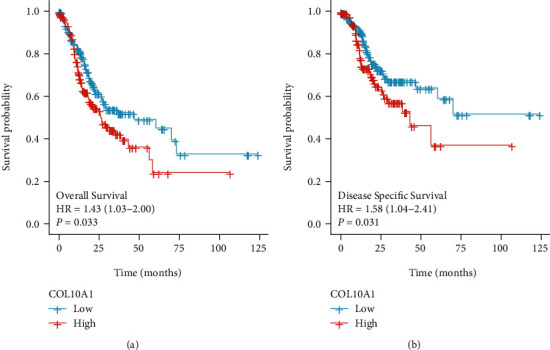
Kaplan-Meier analysis of (a) OS and (b) DSS in 375 GC patients in relation to COL10A1 expression level.

**Figure 5:**
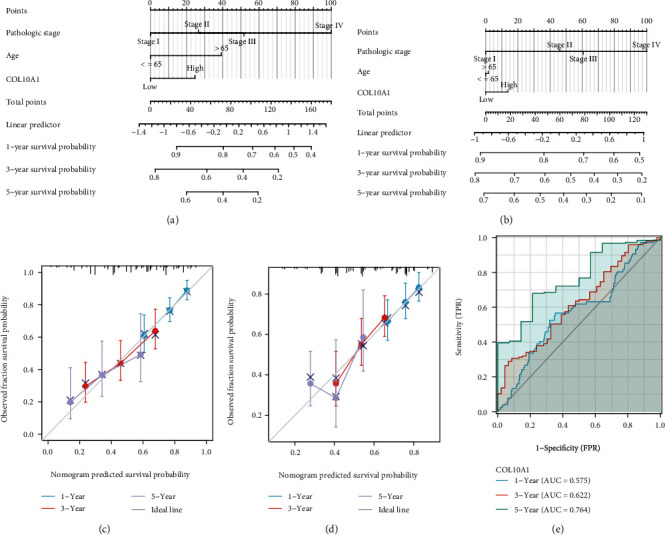
Nomograph of COL10A1 for GC and the time-dependent ROC curve showing the diagnosis value. (a) OS, (b) DSS. (c, d) The calibration curve of nomogram for GC patients in 1 year, 3 years, and 5 years, respectively. (e) The AUC regarding the prediction of 1-, 3-, and 5-year survival rate of GC.

**Figure 6:**
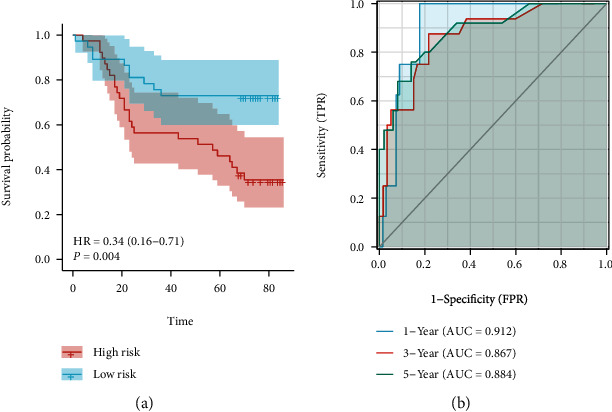
GSE84426 was used to validate the prognosis value of COL10A1. (a) High expression of COL10A1 reported shorter OS. (b) The AUC regarding the prediction of 1-, 3-, and 5-year survival rate of GC.

The R software “survival” package and Kaplan-Meier method together with log-rank test were applied to assess how COL10A1 affected GC patients' overall survival (OS) and disease-specific survival (DSS). We calculated the logarithmic rank *p* value and the 95% CI, followed by plotting a survival curve. The results showed that patients with high COL10A1 expression showed a shorter OS and DSS than those with low COL10A1 expression (Figures 4(a) and 4(b)). Univariate and multivariate Cox regression analyses were applied to investigate whether high COL10A1 expression could independently report poor prognosis of GC patients. Cox univariate survival analysis revealed the important effect of T, N, M, stage, age, and COL10A1 on the OS duration, and multivariate Cox survival analysis showed that age (*p* = 0.001) and COL10A1 (*p* = 0.014) independently predicted a poor prognosis for GC patients (all, *p* < 0.05) (Table 2). Nomograph was built (Figures 5(a)–5(d)), and 1-, 3-, and 5-year AUCs of COL10A1 expression were 0.575, 0.622, and 0.764, respectively, for the survival prediction, that proved the large prognostic value possessed by COL10A1 (Figure 5(e)). GSE84426 was used to validate the prognosis value of COL10A1, and the results were consistent with TCGA (Figures 6(a) and 6(b)).

### 3.4. Interrelation with Tumor-Infiltrating Immune Cells in GC

**Figure 7:**
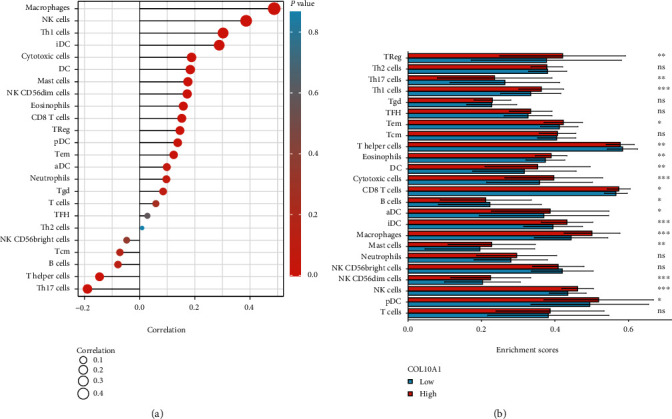
Interrelation with tumor-infiltrating immune cells in GC. (a) ssGSEA. (b) TIMER database. ^∗^*p* < 0.05,  ^∗∗^*p* < 0.01, and^∗∗∗^*p* < 0.001. ns: no significance.

Analysis by the ssGSEA software found the correlation of COL10A1 expression with the tumor-filtrating immune cells, namely, CD8 T cells, cytotoxic cells, DC, eosinophils, iDC, macrophages, mast cells, NK CD56dim cells, NK cells, pDC, T helper cells, Tem, Th1 cells, Th17 cells, and Treg (all *p* < 0.05, Figure 7(a)). Also, the TIMER database found the positive correlation of COL10A1 expression with infiltrating immune cell levels, namely, macrophage, NK, TH1, and iDC cells (Figure 7(b)).

### 3.5. GO, KEGG, and GSEA Analysis of COL10A1 Coexpression-Related Genes

**Figure 8:**
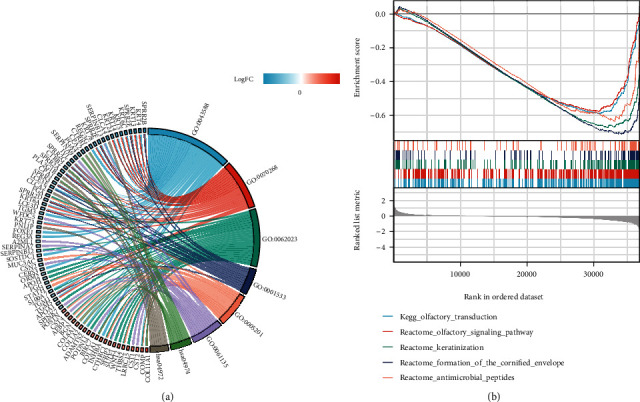
Enrichment analysis of COL10A1 coexpression-related genes. (a) GO assays. (b) GSEA analysis.

Based on the GO analysis, these genes were mainly expressed in the extracellular matrix structural constituent, endopeptidase regulator activity, protein digestion and absorption, and pancreatic secretion (Figure 8(a)). Besides, as revealed by the GSEA analysis, these genes mainly affected the OLFACTORY_TRANSDUCTION, OLFACTORY_SIGNALING_PATHWAY, KERATINIZATION, etc. (Figure 8(b)).

## 4. Discussion

Currently, the commonly used methods for treating GC in early stages are the endoscopic mucosal resection and the endoscopic submucosal dissection [18]. Nevertheless, GC can develop fast and can only be diagnosed at an advanced stage; hence, GC patients have a low 5-year survival rate [19, 20]. Hemotherapy regimens, i.e., SOX (oxaliplatin+S1)/CapeOX (oxaliplatin+capecitabine), FOLFOX (oxaliplatin+leucovorin+5-fluorouracil), and DCF (docetaxel+cisplatin+5-fluorouracil)/DOF (docetaxel+oxaliplatin+5-fluorouracil), mainly serve for GC patients in later stage, which, however, also exhibit limited efficacy. Based on studies, combining the chemotherapy with radiotherapy, surgery, or targeted therapy is treated as the most proper treatment method for improving patient survival, which, however, fails to greatly enhance GC patients' prognosis because chemotherapeutic drugs are toxic; it is hard to screen beneficiaries of targeted therapy drugs; and patients present drug resistance [21, 22].

Based on recent studies, abnormal COL10A1 expression in many cancer types has promoted the tumor growth. Some groups reported the ability of high COL10A1 expression to facilitate GC development in terms of cell proliferation, invasion, and migration. High COL10A1 plasma levels predicted poor OS, which could serve for detecting GC in early stage as a useful biomarker. Huang et al. found the higher COL10A1 expression in colorectal cancer tissues. High COL10A1 expression could cause tumor progression and independently predicted the OS of patients suffering colorectal cancer [23]. As for lung adenocarcinoma, COL10A1 upregulation exhibited positive relation to lymph node metastasis, and COL10A1 was treated as a novel target specific to lung cancer [24]. Breast cancer patients may present less improvement due to the neoadjuvant chemotherapy relative to patients possessing high COL10A1 expression [14]. Our study found the obviously increased COL10A1 expression in GC patients. Importantly, we found that patients with COL10A1 expression showed an advanced clinical stage. It has been known to us that clinical stage can vitally help to determine proper candidates as well as design neoadjuvant treatment strategies specific to advanced tumors. In addition, patients with advanced clinical stage showed a poor prognosis. Thus, our findings suggested that COL10A1 may be associated with the clinical outcome of GC patients. Then, we analyzed survival data using Kaplan-Meier methods, finding that patients who had high COL10A1 expression predicted a shorter OS and DSS relative to patients possessing low COL10A1 expression. Importantly, multivariate Cox survival analysis showed that COL10A1 could independently predict GC patients' poor prognosis. Besides, we obtained a predictive nomogram, which could better predict the COL10A1 expression by virtue of discrimination and calibration. The ROC curve analysis found the better performance exhibited by nomogram relative to other single predictors. Our finding evidenced the advantage of COL10A1 expression in predicting long-term survival as well as stratifying risks.

The immune system can greatly help to eliminate malignant cells inside healthy individuals [25]. However, tumor cells are capable of escaping via immune-mediated infiltration and hence can be hardly cleared by the immune infiltrating cells [26]. Considering the antitumor immunity ability associated with T cells, checkpoint inhibition is commonly applied for clinical cancer immunotherapy [27, 28]. Based on a lot of large clinical trials, immune checkpoint blockade (ICB) therapy could help patients with chemotherapy resistance in EGC and even be a specific agent for palliative treatment [29, 30]. Besides, tumor microenvironment component activity together with related treatment methods may assist in developing combined therapies for ICB [31, 32]. Hence, COL10A1 and immune cells were evaluated with regard to the clinical applicability. In this study, we found the relevance of COL10A1 expression to tumor-filtrating immune cells, namely, CD8 T cells, cytotoxic cells, DC, eosinophils, iDC, macrophages, mast cells, NK CD56dim cells, NK cells, pDC, T helper cells, Tem, Th1 cells, Th17 cells, and Treg. Mast cells and DCs, the first groups of cells in the immune system, are capable of interacting with allergens, other antigens, as well as invading pathogens in the environment. Being in resting states, the two cells cannot play their roles, which may lead to tumor immune escape. Our model was closely related to immunity, finding that COL10A1 expression well reported the immune status regarding the predicted samples.

Undoubtedly, some limitations must be addressed in the present study. Firstly, data in the study are based on public databases; hence, our results shall be validated in vitro and in vivo. Also, COL10A1 exhibited overexpression in tumor tissue from TCGA database relative to normal tissue; hence, COL10A1 expression shall be validated via other studies, such as RT-PCR and Western blot.

## 5. Conclusion

To our knowledge, this is the first study on clinical significance of COL10A1 expression in GC patients. Our study revealed that the expression levels of COL10A1 were upregulated in GC tissues. High expression of COL10A1 predicted poor prognosis for GC. COL10A1 may be useful for evaluating prognosis and added new possibilities for immunotherapy in patients with GC.

## Figures and Tables

**Table 1:** Association between COL10A1 expression and different clinicopathological features of human GC.

Characteristic	Low expression of COL10A1	High expression of COL10A1	*p*
*n*	187	188	
T stage, *n* (%)			<0.001
T1	18 (4.9%)	1 (0.3%)	
T2	44 (12%)	36 (9.8%)	
T3	78 (21.3%)	90 (24.5%)	
T4	45 (12.3%)	55 (15%)	
N stage, *n* (%)			0.901
N0	53 (14.8%)	58 (16.2%)	
N1	51 (14.3%)	46 (12.9%)	
N2	39 (10.9%)	36 (10.1%)	
N3	37 (10.4%)	37 (10.4%)	
M stage, *n* (%)			1.000
M0	166 (46.8%)	164 (46.2%)	
M1	13 (3.7%)	12 (3.4%)	
Pathologic stage, *n* (%)			0.012
Stage I	36 (10.2%)	17 (4.8%)	
Stage II	45 (12.8%)	66 (18.8%)	
Stage III	77 (21.9%)	73 (20.7%)	
Stage IV	19 (5.4%)	19 (5.4%)	
Gender, *n* (%)			0.884
Female	68 (18.1%)	66 (17.6%)	
Male	119 (31.7%)	122 (32.5%)	
Age, *n* (%)			0.899
≤65	82 (22.1%)	82 (22.1%)	
>65	101 (27.2%)	106 (28.6%)	
Histologic grade, *n* (%)			0.095
G1	5 (1.4%)	5 (1.4%)	
G2	77 (21%)	60 (16.4%)	
G3	98 (26.8%)	121 (33.1%)	
H pylori infection, *n* (%)			1.000
No	96 (58.9%)	49 (30.1%)	
Yes	12 (7.4%)	6 (3.7%)	
Barrett's esophagus, *n* (%)			0.612
No	122 (58.7%)	71 (34.1%)	
Yes	11 (5.3%)	4 (1.9%)	
Age, mean ± SD	65.52 ± 10.52	66.13 ± 10.79	0.580

**Table 2:** Prognostic factor for OS of patients with GC determined by using univariate and multivariate COX analysis.

Characteristics	Total (*N*)	Univariate analysis		Multivariate analysis	
		Hazard ratio (95% CI)	*p* value	Hazard ratio (95% CI)	*p* value
T stage	362				
T1&T2	96	Reference			
T3&T4	266	1.719 (1.131-2.612)	0.011	1.189 (0.624-2.264)	0.599
N stage	352				
N0	107	Reference			
N1	97	1.629 (1.001-2.649)	0.049	1.329 (0.672-2.626)	0.413
N2	74	1.655 (0.979-2.797)	0.060	1.502 (0.650-3.469)	0.341
N3	74	2.709 (1.669-4.396)	<0.001	2.142 (0.933-4.917)	0.072
M stage	352				
M0	327	Reference			
M1	25	2.254 (1.295-3.924)	0.004	1.256 (0.534-2.954)	0.602
Pathologic stage	347				
Stage I	50	Reference			
Stage II	110	1.551 (0.782-3.078)	0.209	1.281 (0.474-3.458)	0.626
Stage III	149	2.381 (1.256-4.515)	0.008	1.256 (0.342-4.610)	0.731
Stage IV	38	3.991 (1.944-8.192)	<0.001	2.485 (0.655-9.436)	0.181
Gender	370				
Female	133	Reference			
Male	237	1.267 (0.891-1.804)	0.188		
Age	367				
≤65	163	Reference			
>65	204	1.620 (1.154-2.276)	0.005	1.849 (1.272-2.687)	0.001
COL10A1	370				
Low	185	Reference			
High	185	1.434 (1.030-1.996)	0.033	1.567 (1.096-2.242)	0.014
Histologic grade	361				
G1	10	Reference			
G2	134	1.648 (0.400-6.787)	0.489		
G3	217	2.174 (0.535-8.832)	0.278		

## Data Availability

The data used to support the findings of this study are available from the corresponding authors upon request.
